# Ganoderma Triterpenoids Exert Antiatherogenic Effects in Mice by Alleviating Disturbed Flow-Induced Oxidative Stress and Inflammation

**DOI:** 10.1155/2018/3491703

**Published:** 2018-04-11

**Authors:** Pei-Ling Hsu, Yung-Ching Lin, Hao Ni, Fan-E Mo

**Affiliations:** ^1^Department of Cell Biology and Anatomy, College of Medicine, National Cheng Kung University, Tainan, Taiwan; ^2^Institute of Basic Medical Sciences, College of Medicine, National Cheng Kung University, Tainan, Taiwan

## Abstract

Ganoderma mushrooms, used in traditional Chinese medicine to promote health and longevity, have become widely accepted as herbal supplements. *Ganoderma lucidum* (GL), a commonly seen ganoderma species, is commercially cultivated under controlled conditions for more consistent chemical composition. The medicinal properties of GL are attributable to its antioxidant and anti-inflammatory activities. We intended to assess the effect of GL in atherosclerosis, an arterial condition associated with chronic oxidative stress and inflammation, using a carotid-artery-ligation mouse model. Flow turbulence created in the ligated artery induces oxidative stress and neointimal hyperplasia, a feature of early atherogenesis. Daily oral GL prevented neointimal thickening 2 weeks after ligation. Moreover, the ganoderma triterpenoid (GT) crude extract isolated from GL abolished ligation-induced neointima formation. Mechanistically, endothelial dysfunction was observed 3 days after ligation before any structural changes could be detected. GTs alleviated the oxidative stress and restored the atheroresistent status of endothelium by inhibiting the induction of a series of atherogenic factors, including endothelin-1, von Willebrand factor, and monocyte chemoattractant protein-1 after 3-day ligation. The anti-inflammatory activity of GTs was tested in cultured human umbilical vein endothelial cells (HUVECs) exposed to disturbed flow in an *in vitro* perfusion system. GTs abolished the induction of proinflammatory VCAM-1, TNF-*α*, and IL-6 by oscillatory shear stress. Moreover, the antioxidant activity of GTs was tested in HUVECs against the insult of H_2_O_2_. GTs dissipated the cellular superoxide accumulation imposed by H_2_O_2_, thereby mitigating H_2_O_2_-induced cell damage and proatherogenic response. Our results revealed the atheroprotective properties of ganoderma mushrooms and identified triterpenoids as the critical constituents for those effects. GTs prevent atherogenesis by eliminating disturbed flow-induced oxidative stress and inflammation.

## 1. Introduction

Atherosclerotic disease remains a leading cause of death in the world based on a most recent survey by the World Health Organization. The complication of atherosclerosis gives rise to coronary artery disease leading to myocardial infarction or cerebrovascular disease leading to stroke. The progression of atherosclerosis involves the development of atheromatous plaques in the intima of arteries. The formation of atheroma is accelerated by dysfunctional endothelium via recruiting circulating monocytes and increasing the uptake of low-density lipoproteins (LDLs) [1]. Monocytes transmigrate into the intima and differentiate into macrophages. The macrophages ingest oxidized LDL and become foam cells. Oxidized lipoproteins and fatty acids trigger a sustained oxidative burst and apoptosis of foam cells, which leads to the formation of atheroma consisting of a lipid core, apoptotic cells, debris, and many inflammatory cells [2]. The dysfunction of the endothelium, in the context of atherosclerotic cardiovascular disease, encompasses a wide range of maladaptation in its normal functional phenotypes for the regulation of thrombosis, local vascular tone, redox balance, and inflammatory response [1].

Endothelial dysfunction can be induced by inflammatory cytokines, oxidative stress, hypertension, hypercholesterolemia, and diabetes [1]. Though these risk factors exist in the entire arterial system, atherosclerosis preferentially develops at arterial branches or curvatures, where the local flow is disturbed [3]. Disturbed flow imposes low and oscillatory sheer stress, which downregulates the nitric oxide production and causes other maladaptive alterations in endothelial functional phenotype [3–5]. Current therapy for atherosclerosis primarily targets hypercholesterolemia, thrombosis, or inflammation, however, lacks a good strategy for restoring endothelial function under disturbed flow. Here, we used a disturbed flow-induced atherogenic mouse model by ligating the carotid artery to evaluate potential new treatments.

Ganoderma mushrooms (Lingzhi in Chinese) are a traditional Chinese herbal medicine that have been widely accepted as a nutritional supplement. Among many species of the mushrooms, *Ganoderma lucidum* (GL) is most commonly seen and is commercially cultivated under controlled conditions to obtain mushrooms with more consistent chemical composition. GL possesses antihypertensive and hypocholesterolemic activities among other medicinal benefits [6]. The primary bioactive compounds in GL include triterpenoids and polysaccharides [6]. GL prevents cardiac damage in animal models by alleviating the oxidative stress associated with myocardial injury [7]. The triterpenoid fraction of ganoderma, consisting of more than 300 lanostane-tetracyclic compounds [8], provides antioxidant activities to prevent myocardial injury [9]. Ganoderma triterpenoids (GTs) also suppress inflammatory response [10] by directly scavenging the free radicals or systemically enhancing the antioxidant enzymes [11], thereby lowering lipid peroxidation in mice [12]. Inflammation and oxidized LDL are two major risk factors driving the progression of atherosclerosis. We intended to evaluate the atheroprotective activities of GL and its triterpenoid constituents using the carotid-artery-ligation mouse model.

## 2. Materials and Methods

### 2.1. Animals

**Figure 1:**
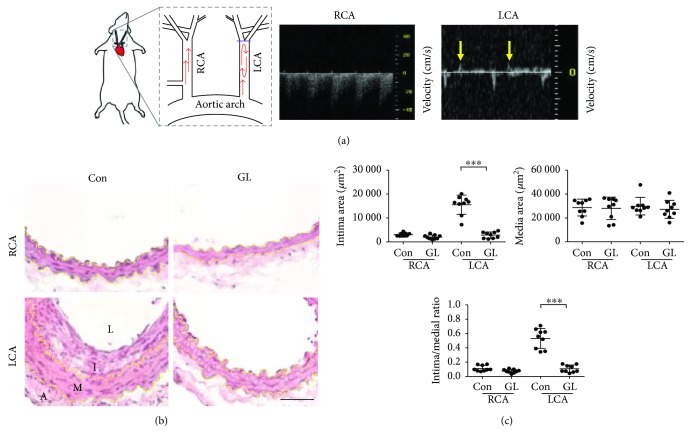
*Ganoderma lucidum* (GL) protected the carotid artery from disturbed flow-induced atherogenesis. (a) The diagram illustrates the carotid-artery-ligation model. A purple bar indicates the blockage in the ligated left common carotid artery (LCA). Oscillatory blood flow was generated by arterial ligation. Yellow arrows indicate the flow recirculation in the ligated LCA observed in the pulsed wave Doppler image. The unligated right common carotid artery (RCA) served as the sham control. (b) Carotid arteries were excised 14 days after ligation from mice fed with GL (300 mg/kg/day) or water vehicle control (Con) (*n* = 9 for each group) and processed for H&E staining. Yellow dashed lines delineate the internal or external elastic lamellae. A: adventitia; I: intima; L: lumen; M: media. Bar: 50 *μ*m. (c) The intima and media areas of each arterial tissue section were quantified using the Nikon NIS-Elements D program. Data are means ± SEM from 6 to 8 serial sections from each artery. Statistical significance was calculated using two-way ANOVA and post hoc Tukey's tests, ^∗∗∗^*p* < 0.001.

This study was carried out in accordance with the recommendations of the *Guide for the Care and Use of Laboratory Animals* published by the United States National Institutes of Health. All animal use protocols were approved by the Institutional Animal Care and Use Committee of the National Cheng Kung University. The animal sample size (*n*) was estimated by a power analysis of the pilot study using the G^∗^Power program. We first tested the atheroprotective property of GL in a disturbed flow-induced atherogenesis mouse model. We followed by identifying the constituents contributing to the atheroprotective property of GL by using GT extracts. Furthermore, we tested deferred GTs treatment to assess the potential for using GT to treat existing conditions. Mice were anesthetized with chloral hydrate (300 mg/kg; i.p.) before surgery. If pain or distress was observed after surgery, nalbuphine (1.2 mg/kg; s.c.) was administered to the mice. Male BALB/c mice between 2 and 5 months old were used in this study. To induce neointima formation, a ligature was made at the end of the left common carotid artery (LCA) near the carotid bifurcation (Figure 1(a)). Granules of GL (300 mg/kg/day Shuang Hor Superfine Lingzhi; Double Crane, Taiwan) derived from concentrated aqueous-ethanolic extract of the fruiting body of GL suspended in water or water vehicle control were delivered orally via a gavage needle 2 h before ligation and daily for the remainder of the waiting period. Alternatively, GTs (300 mg/kg/day; a generous gift from the Biotechnology Research and Development Institute of Double Crane Group, Taiwan) or DMSO vehicle control was subcutaneously injected immediately after ligation and daily for the remainder of the waiting period. Blood flow was monitored with pulsed wave Doppler imaging using the VisualSonics Vevo 770 with a 40 MHz probe.

### 2.2. Histological Analysis

Mice were euthanized 3, 14, or 17 days after surgery. LCAs and right common carotid arteries (RCAs) were harvested and cryopreserved in OCT compound. Eight micrometer cryosections of the artery tissue from the segment between 1.5 and 2 mm from the ligature were stained with hematoxylin and eosin (H&E) or TUNEL staining (Millipore). For immunofluorescence staining, sections of arterial tissue were incubated with the antibodies as indicated, including anti-CD31 (Abcam ab28364, 1 : 50), anti-F4/80 (Abcam ab6640, 1 : 100), anti-endothelin-1 (ET-1) (Abcam ab2786, 1 : 250), anti-von Willebrand factor (vWF) (Abcam ab68545, 1 : 50), and anti-monocyte chemoattractant protein-1 (MCP-1) (Abcam ab8101, 1 : 20). The number of cells displaying specific staining was scored in a blinded manner.

### 2.3. Dihydroethidium (DHE) Staining

DHE after being oxidized by superoxides intercalates into DNA and generates red fluorescence. Eight serial arterial sections were incubated with DHE (2 *μ*M) at 37°C for 30 min and then immunostained for endothelial-specific CD31 (green) and counterstained with DAPI (blue) for nuclei. The numbers of DHE^+^ and total endothelial cells on each artery section were scored in a blinded manner.

### 2.4. Cell Culture

Human umbilical vein endothelial cells (HUVECs) were purchased from the Bioresource Collection and Research Center (Hsinchu, Taiwan). Cells were cultured on gelatin-coated plates in medium 199 (Gibco) supplemented with 10% FBS, 25 U/ml heparin (Sigma), 30 *μ*g/ml endothelial cell growth supplement (Millipore), 1x GlutaMax (Gibco), and 1.5 g/l sodium bicarbonate at 37°C and 5% CO_2_.

### 2.5. Annexin V/Propidium Iodide (PI) Staining for Apoptosis

HUVECs were pretreated with GTs (500 *μ*g/ml) or DMSO vehicle control for 1 h before H_2_O_2_ (400 *μ*M) was added, and incubated for additional 24 h. Treated cells were harvested and incubated with an FITC-conjugated annexin V antibody (BD Biosciences 556419, 1 : 20) and PI at room temperature for 15 min before fluorescence was measured by flow cytometry. The FlowJo software was used to analyze the results. The concentration of 500 *μ*g/ml GTs was determined in a dose-response assessment of GTs against H_2_O_2_-induced apoptosis by counting nuclear condensation after DAPI staining (data not shown).

### 2.6. Reactive Oxygen Species (ROS) Measurements

HUVECs were treated with H_2_O_2_ (400 *μ*M) in the presence of or no GTs (500 *μ*g/ml, 1 h pretreatment) for 30 min. To measure cytosolic ROS, live cells were loaded with the ROS dye CM-H2DCFDA (5 *μ*M) 15 min prior to the end of H_2_O_2_ treatment. Cells were then harvested and resuspended in cold PBS containing 5% FBS. Fluorescence was measured by flow cytometry and analyzed using the FlowJo software.

### 2.7. Immunocytochemistry

HUVECs were treated with H_2_O_2_ (400 *μ*M) in the presence of or no GTs (500 *μ*g/ml, 1 h pretreatment) for 5 h. Treated cells were fixed and permeabilized with 0.1% Tween 20 in PBS washing buffer for 30 min at room temperature. Cells were then stained with anti-ET-1 (Abcam ab2786, 1 : 200) or anti-MCP-1 (Abcam ab8101, 1 : 200) antibodies.

### 2.8. *In Vitro* Perfusion System for Simulation of Blood Flow

HUVECs seeded on microslides (ibidi 80186) were perfused with unidirectional flow to generate laminar shear stress (LSS, 12 dyn/cm^2^), or oscillating flow to generate oscillatory shear stress (OSS, ±5 dyn/cm^2^), or static control for 24 h using the ibidi pump system in the presence of or no GTs (500 *μ*g/ml). Total RNA was extracted from treated cells and was subjected to quantitative RT-PCR using the Applied Biosystems StepOne Real-Time PCR Systems. The following gene-specific primer sets were used: (1) Il-6: 5′-GGACGGCTTTTACTTAAACGCCAAGG-3′ (sense) and 5′-ATCTTCCCTAGTTACCCAGGTTCAGC-3′ (antisense); (2) Tnf*α*: 5′-AAGAGTTCCCCAGGGACCTCT-3′ (sense) and 5′-CCTGGGAGTAGATGAGGTACA-3′ (antisense); (3) Vcam-1: 5′-CATTGACTTGCAGCACCACA-3′ (sense) and 5′-AGATGTGGTCCCCTCATTCG-3′ (antisense); (4) Gapdh: 5′-GAAGGTGAAGGTCGGAGTC-3′ (sense) and 5′-GAAGATGGTGATGGGATTTC-3′ (antisense). The amplification conditions were 10 min at 95°C, 40 cycles of 10 sec/95°C–1 min/60°C. The expression values of individual genes were normalized to Gapdh using the comparative cycle threshold method (2^−ΔΔCT^).

### 2.9. Statistical Analysis

All assays were repeated at least 3 times and yielded similar patterns. Values are means ± SEM. Comparisons were made using two-way ANOVA and post hoc Tukey's tests. Significance was set at *p* < 0.05 and indicated as ^∗^*p* < 0.05; ^∗∗^*p* < 0.01; or ^∗∗∗^*p* < 0.001.

## 3. Results

### 3.1. GL Protected the Carotid Artery from Disturbed Flow-Induced Atherogenesis

We evaluated the effect of GL using a disturbed flow-induced atherogenic mouse model by carotid artery ligation. The blockage of the LCA after ligation was confirmed with pulsed wave Doppler imaging (Figure 1(a)). A unidirectional (away from the heart) pulsatile blood flow pattern was detected in the unligated RCA (Figure 1(a)). By contrast, the flow rate was much reduced after ligation in the LCA and flow recirculation (back to the heart displayed as positive velocities) was detected (Figure 1(a), arrows). The straight part of the healthy common carotid arteries is resistant to atherogenesis. However, low/reciprocating flow shear stress created by the ligation induces intimal hyperplasia, thereby the formation of neointima (Figure 1(b)). To test the atheroprotectivity of GL, mice were treated with oral GL (300 mg/kg/day) or water vehicle control after LCA ligation (*n* = 9 for each group). The unligated RCA was used as a sham control. Both intima areas and intima/media ratios in the LCA were increased 14 days after ligation in the control (Con) mice (Figure 1(c)). Media layers were not affected by the ligation. Remarkably, GL-treated mice were resistant to ligation-induced intimal hyperplasia (Figures 1(b) and 1(c)), demonstrating the atheroprotective property of GL.

### 3.2. GTs Prevented Carotid Artery Ligation-Induced Neointima Formation

**Figure 2:**
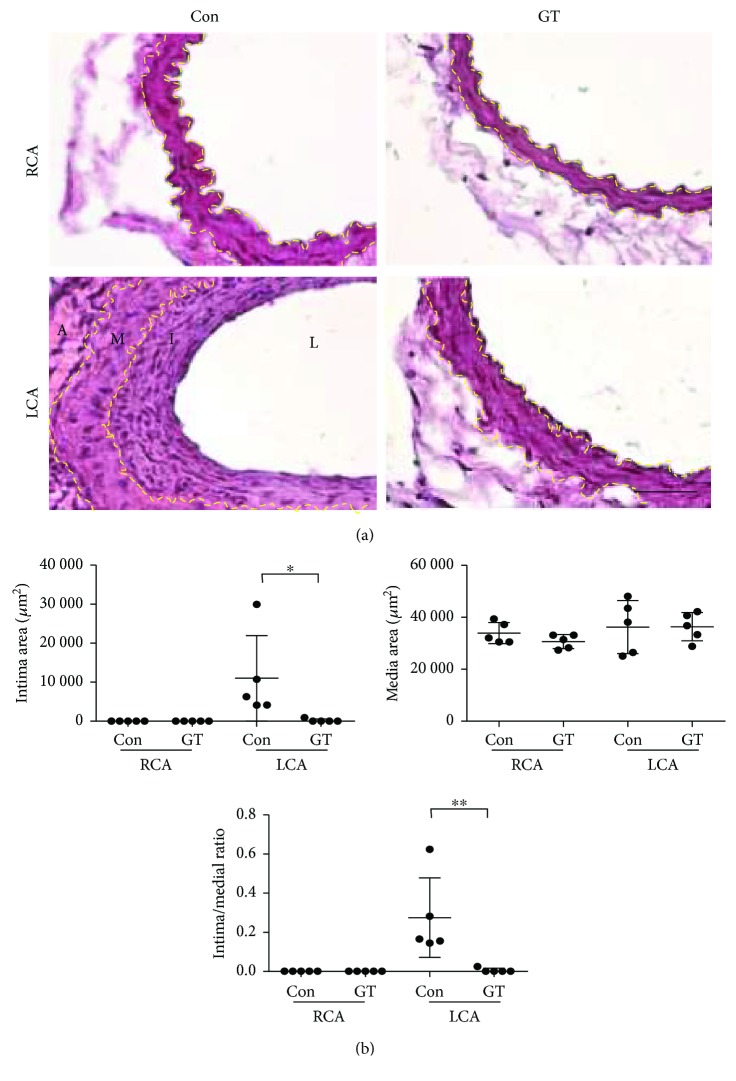
Ganoderma triterpenoids (GTs) prevented neointimal hyperplasia after carotid artery ligation. (a) LCAs were excised 14 days after ligation from mice receiving subcutaneous injections of GTs (300 mg/kg/day) or DMSO vehicle control (Con) (*n* = 5 for each group) immediately after ligation and processed for H&E staining. Unligated RCAs served as the sham control. Yellow dashed lines delineate the internal or external elastic lamellae. A: adventitia; I: intima; L: lumen; M: media. Bar: 50 *μ*m. (b) The intima and media areas were quantified as described in Figure 1. Data are means ± SEM from 6 to 8 serial sections from each artery (*n* = 5). Statistical significance was calculated using two-way ANOVA and post hoc Tukey's tests, ^∗^*p* < 0.05, ^∗∗^*p* < 0.01.

**Figure 3:**
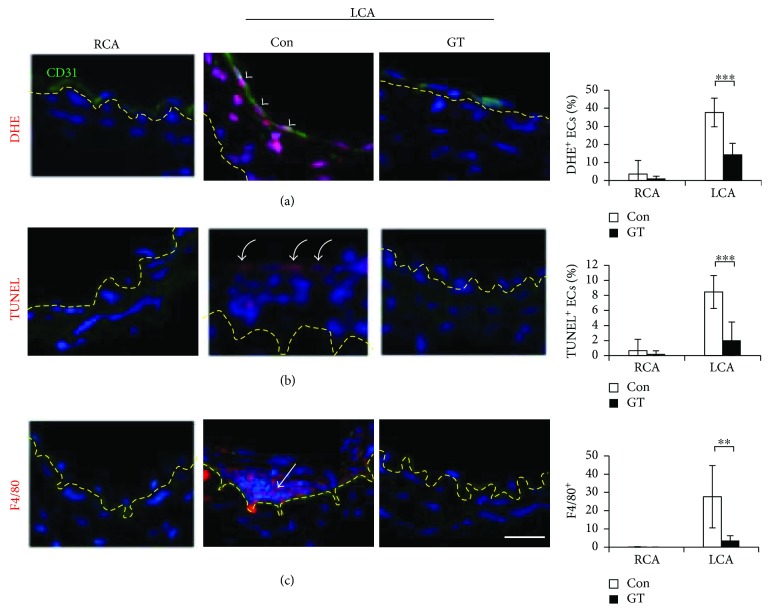
GTs inhibited atherogenesis in the ligated artery. Arterial sections, collected from mice treated with GTs (300 mg/kg/day; s.c.) or DMSO vehicle control (Con) 14 days after ligation as described in Figure 2, were stained with the superoxide indicator dihydroethidium (DHE, red in (a)), TUNEL (for apoptosis, red in (b)), an anti-CD31 (an endothelial cell marker, green) antibody, or an anti-F4/80 antibody (a monocyte/macrophage marker, red in (c)), and counterstained with DAPI for nuclei. Yellow dashed lines delineate the internal elastic lamellae. The tissue above the yellow dashed line is the intima. (a) Arrowheads indicate the superoxide-accumulated endothelial cells (DHE^+^/CD31^+^ ECs with white nuclei) in the Con LCA. Numbers are the percentage of DHE^+^ ECs. (b) Curved arrows indicate the apoptotic ECs (TUNEL^+^, inner lining cells with pink nuclei). Numbers are the percentage of TUNEL^+^ ECs. (c) An arrow indicates the infiltrating macrophages (F4/80^+^, pink nuclei) in the intima. Numbers are the F4/80^+^ cells in the intima of each arterial section. All the numbers in (a), (b), and (c) are means ± SEM from 8 (a, b) or 4 (c) serial sections from each artery (*n* = 5 for each group). Bar: 25 *μ*m. Statistical significance was calculated using two-way ANOVA and post hoc Tukey's tests, ^∗∗^*p* < 0.01, ^∗∗∗^*p* < 0.001.

To test the effect of GTs in mice, we used the crude triterpenoids isolated from the acidic ethyl acetate-soluble material of the fruiting body of GL, which consists of >90% of total triterpenoid compounds, including ganoderic acids A (21%), B (8%), C (4%), C5 (3%), C6 (1%), D (10%), E (2%), G (5.5%), and ganoderenic acid D (7.5%), in addition to other minor triterpenoid components analyzed with the reverse phase HPLC fingerprinting as previously described [13]. Mice were subcutaneously injected with GTs (300 mg/kg/day) or vehicle control DMSO (*n* = 5 for each group). Subcutaneous administration of GTs dissolved in DMSO was more effective than oral delivery in our initial testing (data not shown). GT-treated mice displayed similar resistance against ligation-induced intimal thickening (Figures 2(a) and 2(b)) as observed in mice receiving GL. The oxidative stress, identified with a superoxide indicator (DHE^+^, red, arrowheads), in the endothelium (CD31^+^, green) of the LCA was ameliorated by GTs (Figure 3(a)). Furthermore, the endothelial apoptosis (TUNEL^+^, red, curved arrows) in the LCA was abolished by GTs (Figure 3(b)). The recruitment of monocytes/macrophages (F4/80^+^, red, arrows) to the neointima was increased by ligation, and was eliminated by GTs (Figure 3(c)), demonstrating the anti-inflammatory benefit provided by GTs.

### 3.3. GTs Alleviated Disturbed Flow-Induced Oxidative Stress and Proatherogenic Response in Endothelial Cells

**Figure 4:**
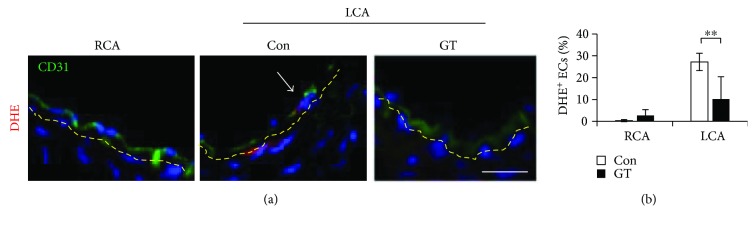
GTs alleviated the initial oxidative stress in the endothelium after ligation.(a) LCA and RCA from mice receiving Con (DMSO) or GT (300 mg/kg/day; s.c.) treatment were excised 3 days after ligation before any structural change occurred. Arterial tissue sections were processed for DHE staining. An arrow indicates a DHE^+^ (red)/CD31^+^ (green) EC. Yellow dashed lines delineate the internal elastic lamellae. Bar: 25 *μ*m. (b) The percentages of DHE^+^ ECs are means ± SEM from 8 serial sections from each artery (*n* = 5 for each group). Statistical significance was calculated using two-way ANOVA and post hoc Tukey's tests, ^∗∗^*p* < 0.01.

**Figure 5:**
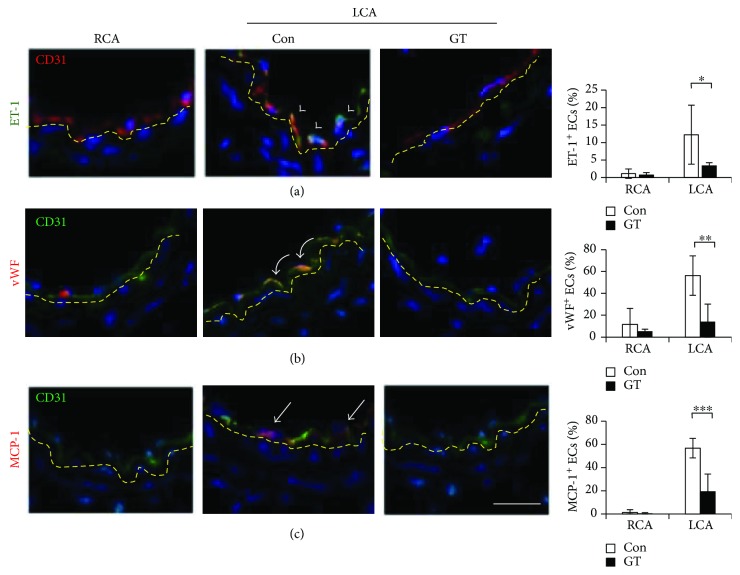
GTs preserved the atheroprotective property of endothelial cells in the ligated artery. LCA and RCA tissue sections, collected 3 days after ligation from mice receiving Con (DMSO) or GT (300 mg/kg/day; s.c.) treatment immediately after ligation and daily for the remainder of waiting period, were immunostained for proatherogenic factors ET-1, vWF, or MCP-1 as indicated. (a) Arrowheads indicate the ET-1^+^ (green)/CD31^+^ (red) ECs. (b) Curved arrows indicate the vWF^+^ (red)/CD31^+^ (green) ECs. (c) Arrows indicate the MCP-1^+^ (red)/CD31^+^ (green) ECs. The percentages of the positive ECs are means ± SEM from 4 serial sections from each artery (*n* = 5 for each group). Statistical significance was calculated using two-way ANOVA and post hoc Tukey's tests, ^∗^*p* < 0.05, ^∗∗^*p* < 0.01, ^∗∗∗^*p* < 0.001. Yellow dashed lines delineate the internal elastic lamellae. Bar: 25 *μ*m.

Alteration in endothelial function precedes the structural changes in atherogenesis [1]. We investigated the effect of GTs in regulating endothelial function in mice 3 days after ligation before any structural changes could be detected. We found that oxidative stress was elevated in the intima of the LCA and ~25% of the endothelial cells (CD31^+^, green) were DHE^+^ (red) (Figure 4, arrow). Sustained oxidative stress leads to endothelial dysfunction and the induction of atherogenic factors [3]. Indeed, ET-1 (green in Figure 5(a), arrowheads), vWF (red in Figure 5(b), curved arrows), and MCP-1 (red in Figure 5(c), arrows) were induced in the endothelium of the control LCA 3 days after ligation. ET-1 induces vasoconstriction and is overexpressed in atherosclerosis [14]. vWF triggers thrombosis by mediating the initial adhesion of platelets at sites of vascular injury [15]. MCP-1 promotes vascular inflammation by recruiting circulating monocytes to the lesion sites [16]. The induction of ET-1, vWF, and MCP-1 was suppressed in the LCA of GT-treated mice (Figure 5). Together, these results indicated that GTs ameliorate the disturbed flow-induced oxidative stress and proatherogenic response to prevent the progression of atherogenesis.

### 3.4. GTs Suppressed Oscillating Flow-Induced Inflammatory Response in Endothelial Cells

**Figure 6:**
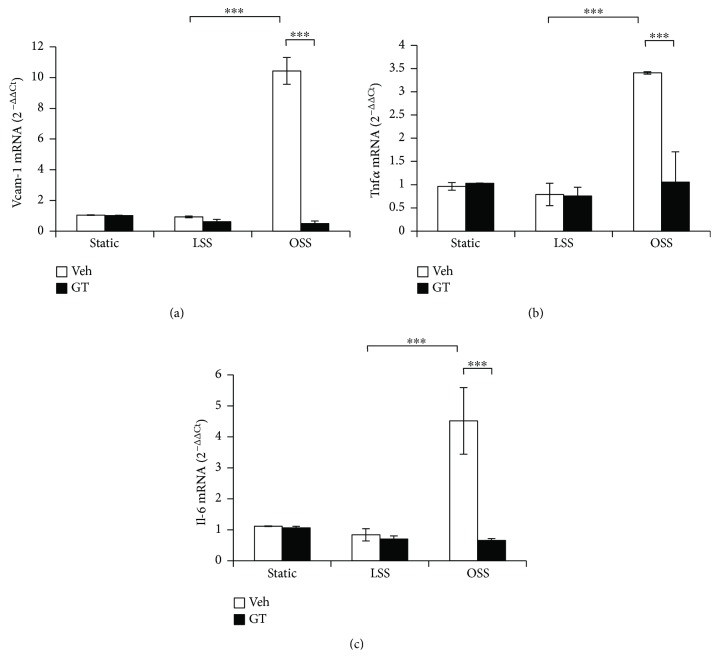
GTs suppressed disturbed flow-induced inflammatory response in ECs. Human umbilical vein endothelial cells (HUVECs) cultured on *μ*-slides were exposed to laminar shear stress (LSS, 12 dyn/cm^2^), oscillatory shear stress (OSS, ±5 dyn/cm^2^), or static control for 24 h in the presence of GTs (500 *μ*g/ml) or DMSO vehicle control (Veh). mRNA from treated cells was isolated and examined for the expression levels of proinflammatory genes (a) Vcam-1, (b) Tnf*α*, or (c) Il-6 using quantitative RT-PCR. Data are shown as mean ± SEM of triplicate experiments. Statistical significance was calculated using two-way ANOVA and post hoc Tukey's tests, ^∗∗∗^*p* < 0.001.

To test the anti-inflammatory activity of GTs directly, HUVECs were exposed to unidirectional laminar flow (LSS) or slower and oscillating flow (OSS) in an *in vitro* perfusion system for 24 h to simulate different hemodynamic patterns in blood vessels. We then used qRT-PCR to measure the expression of a set of proinflammatory genes in the treated cells. We found that OSS upregulated the expression of vascular cell adhesion molecule- (VCAM-) 1 (Figure 6(a)), tumor necrotic factor- (TNF-) *α* (Figure 6(b)), and interleukin- (IL-) 6 (Figure 6(c)), compared with the levels in cells under LSS or static control. GTs (500 *μ*g/ml) abrogated the induction of VCAM-1, TNF-*α*, and IL-6 by OSS (Figure 6), demonstrating the anti-inflammatory activity of GTs.

### 3.5. GTs Protected Endothelial Cells against Oxidative Insults

**Figure 7:**
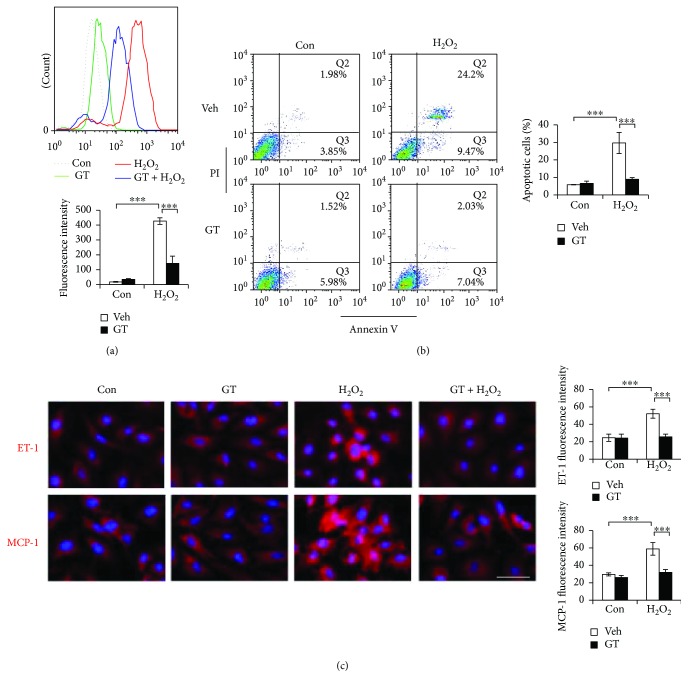
GTs ameliorated H_2_O_2_-induced oxidative stress in the ECs. (a) HUVECs were preincubated with GTs (500 *μ*g/ml) or vehicle (Veh) control DMSO for 1 h before treated with or without H_2_O_2_ (400 *μ*M, 30 min). Cells were loaded with the fluorescent dye DCFDA for measuring the levels of reactive oxygen species (ROS) using flow cytometry. The upper panel shows a representative histogram overlay of the ROS fluorescent intensities. Quantitative fluorescence intensities are means ± SEM of triplicate experiments. (b) Cells were preincubated with GTs (500 *μ*g/ml) or Veh (DMSO) for 1 h before treated with or without H_2_O_2_ (400 *μ*M) for 24 h. Cells were then stained with annexin V/PI and analyzed for apoptosis. The percentage of the apoptotic cells includes the numbers shown in the lower right Q3 (early apoptosis) and in the upper right Q2 (late apoptosis) quadrants. Data are means ± SEM of triplicate experiments. (c) HUVECs pretreated with GTs (500 *μ*g/ml, 1 h) or Veh (DMSO) were treated with or without H_2_O_2_ (400 *μ*M) for 5 h, and followed by immunostaining with anti-ET-1 (red) or anti-MCP-1 (red) antibodies as indicated, and counterstaining with DAPI. The fluorescence intensity was quantified using the NIH ImageJ program. Data are means ± SEM of triplicate experiments. Bar: 25 *μ*m. Statistical significance was calculated using two-way ANOVA and post hoc Tukey's tests, ^∗∗∗^*p* < 0.001.

To test the antioxidant activity of GTs, we challenged HUVECs with H_2_O_2_ and measured their cellular ROS levels after 30 min H_2_O_2_ treatment (400 *μ*M) with or without GTs (500 *μ*g/ml, 1 h pretreatment). The induction of intracellular ROS by H_2_O_2_ was significantly reduced by GTs (Figure 7(a)). Consequently, H_2_O_2_-induced (400 *μ*M, 24 h) apoptosis was abolished by GTs (500 *μ*g/ml, 1 h pretreatment) in HUVECs. Apoptosis was measured with flow cytometry after annexin V/PI staining. Cells in the early (lower right quadrants, Q3) or late (upper right quadrants, Q2) stages of apoptosis were scored (Figure 7(b)). Oxidative stress leads to endothelial cell dysfunction prior to apoptosis. We found that both ET-1 (red in Figure 7(c)) and MCP-1 (red in Figure 7(c)) intensities were elevated by 5 h H_2_O_2_ treatment (400 *μ*M). The induction of atherogenic ET-1 and MCP-1 by H_2_O_2_ was abolished by GTs (Figure 7(c)), suggesting that the atheroprotective property of endothelial cells was preserved by GTs attributable to their antioxidant activity.

### 3.6. Deferred GT Treatment Effectively Inhibited Atherogenesis

**Figure 8:**
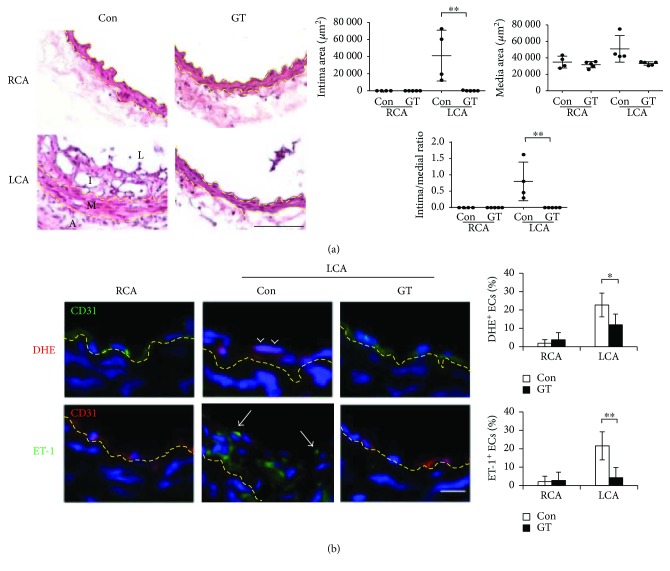
Deferred GT treatment dissipated the oxidative stress and ET-1 induction in endothelial cells and prevented neointima formation in the ligated arteries. GT (300 mg/kg/day; s.c.; *n* = 5) or Con (DMSO; *n* = 4) treatment was deferred for 3 days after ligation and continued for additional 14 days before the arteries were excised and analyzed. (a) LCA and RCA were processed for H&E staining. The quantitation of intima and media areas was as described in Figure 1. Data are means ± SEM from 6 to 8 serial sections from each artery. (b) Arterial tissue sections were subjected to DHE (red) staining, or ET-1 (green) immunostaining, in addition to CD31 immunostaining (green or red as indicated) and DAPI counterstaining. Arrow heads indicate the DHE^+^ ECs with pink nuclei. Arrows indicate the green ET-1^+^ ECs. Quantitation was done as described in Figure 1. The percentages of the positive ECs are means ± SEM from 8 (DHE) or 4 (ET-1) serial sections from each artery. Statistical significance was calculated using two-way ANOVA and post hoc Tukey's tests, ^∗^*p* < 0.05, ^∗∗^*p* < 0.01. Yellow dashed lines delineate the internal or external elastic lamellae. Bars in (a) 100 *μ*m and in (b) 25 *μ*m.

To assess the therapeutic potential of GTs on preexisting conditions, GT treatment was deferred for 3 days after ligation when endothelial function had been compromised. We examined the arteries 14 days after the start of GT treatment and found that deferred GT treatment effectively blocked neointima formation (Figure 8(a)). Deferred GT treatment also alleviated the oxidative stress (DHE^+^, arrowheads in Figure 8(b)) and suppressed the endothelial induction of ET-1 (arrows in Figure 8(b)) in the LCA. These findings suggested that GTs restored endothelial cells from acquired proatherogenic phenotype after ligation and reversed the progression of atherogenesis, reinforcing the therapeutic properties of GTs.

## 4. Discussion

Ganoderma mushrooms have long been used in traditional Chinese medicine. Because of its long-term safety and tolerance, ganoderma is widely accepted as a nutritional supplement in the world. GL has a diverse blend of medicinal properties [6]. In particular, the antihypertensive, antiplatelet aggregation, and hypocholesterolemic properties of GL directly counteract many of the major atherogenic risk factors. Here we tested a new atheroprotective effect of GL using the carotid-artery-ligation mouse model. The ligation of the artery generates disturbed blood flow, a critical atherogenic factor currently with no cure. We found that GL protected arteries from disturbed flow-induced atherogenesis and the triterpenoid fraction is the critical constituents for these effects. GTs alleviated oxidative stress and inflammation, thereby preventing neointimal hyperplasia in the ligated arteries.

For atherosclerosis studies, the hyperlipidemic *apolipoprotein E*-null (*ApoE*^−/−^) mice on a high-fat diet are frequently used to generate vascular lesions. Here we used wild-type mice on a regular diet to avoid the complication involving LDL and to focus on the regulation of endothelial function by flow shear stress. Our finding herein defines a novel atheroprotective activity of GL directly on arterial endothelial cells independent of its lipid-lowering property. Without hyperlipidemia, neointimal hyperplasia is induced by arterial ligation, representing the initial feature of atherogenesis. To further develop the complete clinical feature of atherosclerotic lesions, such as lipid deposits and necrotic cores, *ApoE*^−/−^ mice (on a regular diet) can be used in the carotid-artery-ligation model to generate atherosclerotic plaques [17]. Our findings here support a further study to test the atheroprotective characteristics of GL against disturbed flow under hyperlipidemic conditions in *ApoE*^−/−^ mice.

The atheroprotective effect of GTs is attributable to their anti-inflammatory and antioxidant activities, suggested by the reduction of macrophage infiltration and oxidative stress in the ligated arteries of GT-treated mice. More direct evidence was shown in the *in vitro* assays, in which GTs inhibited the inflammatory response by OSS and reduced the cellular ROS accumulation and the induction of ET-1 and MCP-1 by H_2_O_2_ in HUVECs. The anti-inflammatory and antioxidant activities of GTs maintain endothelial function under stressed conditions, thereby promoting atheroresistence, anti-inflammation, and antithrombosis via downregulating ET-1, MCP-1, and vWF. These atheroprotective effects require separate medications according to current clinical treatment regimens. Furthermore, by promoting vascular health and not interfering with systemic physiology, GTs can be used with less potential side effects, such as the antithrombotic therapy-associated bleeding risks. The effectiveness of the deferred GT treatment indicates that GTs can reverse preexisting endothelial conditions and restore the atheroresistent status of endothelial cells, further strengthening the therapeutic use of GTs. Patients with the conditions, such as existing atherosclerotic plaque, in-stent restenosis, bypass graft occlusion, transplant vasculopathy, or aortic valve calcification, are prone to further development of atherosclerotic lesion due to endothelial dysfunction caused by disturbed blood flow in the affected vascular segments [3]. GTs may promote normal endothelial function and prevent atherosclerosis in these high-risk patients.

Though more than 316 GTs have been identified [8], the triterpenoid constituents of natural ganoderma mushrooms are in low abundance (<3%) [13]. Nonetheless, the most abundant triterpenoids, including ganoderic acids A, B, C, and D, have been demonstrated for their antioxidant properties [18]. Accumulating evidence on the medicinal properties of GTs has promoted the advancement in the industrialized GT isolation technique [19], which allows the development of triterpenoid-enriched ganoderma extracts. Certain over-the-counter ganoderma extracts contain more than 30% of triterpenoid contents based on manufacturer data. It is worth noting that the relatively poor bioavailability of GTs via oral administration can be improved by preconjugating with solid lipid nanoparticles [20]. Therefore, solid lipid nanoparticles can be used to facilitate the absorption of GTs into the circulation to treat atherosclerosis.

Our findings provide evidence that triterpenoids contribute to the atheroprotection of ganoderma and can be used to enhance the efficacy of ganoderma supplements. Nonetheless, the involvement of other constituents, such as polysaccharides, is not excluded by this study. We are currently testing the correlation between triterpenoid contents and the atheroprotective efficacy of ganoderma extracts by comparing a series of ganoderma products with different levels of triterpenoid contents using the carotid-artery-ligation mouse model.
